# Dual Contrast - Magnetic Resonance Fingerprinting (DC-MRF): A Platform for Simultaneous Quantification of Multiple MRI Contrast Agents

**DOI:** 10.1038/s41598-017-08762-9

**Published:** 2017-08-16

**Authors:** Christian E. Anderson, Shannon B. Donnola, Yun Jiang, Joshua Batesole, Rebecca Darrah, Mitchell L. Drumm, Susann M. Brady-Kalnay, Nicole F. Steinmetz, Xin Yu, Mark A. Griswold, Chris A. Flask

**Affiliations:** 1 Department of Radiology, Case Western Reserve University, Cleveland, OH USA; 2 Department of Biomedical Engineering, Case Western Reserve University, Cleveland, OH USA; 3 Frances Payne Bolton School of Nursing, Case Western Reserve University, Cleveland, OH USA; 4 Department of Genetics and Genome Sciences, Case Western Reserve University, Cleveland, OH USA; 5 Department of Pediatrics, Case Western Reserve University, Cleveland, OH USA; 6 Department of Molecular Biology and Microbiology, Case Western Reserve University, Cleveland, OH USA; 7 Department of Neurosciences, Case Western Reserve University, Cleveland, OH USA; 8 Department of Materials Science and Engineering, Case Western Reserve University, Cleveland, OH USA; 9 Department of Macromolecular Science and Engineering, Case Western Reserve University, Cleveland, OH USA; 10 Division of General Medicine-Oncology, Case Western Reserve University, Cleveland, OH USA; 11 Department of Physiology and Biophysics, Case Western Reserve University, Cleveland, OH USA

## Abstract

Injectable Magnetic Resonance Imaging (MRI) contrast agents have been widely used to provide critical assessments of disease for both clinical and basic science imaging research studies. The scope of available MRI contrast agents has expanded over the years with the emergence of molecular imaging contrast agents specifically targeted to biological markers. Unfortunately, synergistic application of more than a single molecular contrast agent has been limited by MRI’s ability to only dynamically measure a single agent at a time. In this study, a new Dual Contrast - Magnetic Resonance Fingerprinting (DC – MRF) methodology is described that can detect and independently quantify the local concentration of multiple MRI contrast agents following simultaneous administration. This “multi-color” MRI methodology provides the opportunity to monitor multiple molecular species simultaneously and provides a practical, quantitative imaging framework for the eventual clinical translation of molecular imaging contrast agents.

## Introduction

Over the past 3 decades, Magnetic Resonance Imaging (MRI) has become an essential medical imaging modality due to its exceptional soft tissue contrast and lack of ionizing radiation. Along with a wide variety of endogenous tissue contrast mechanisms, many MRI applications utilize an intravenous injection of an MRI contrast agent (e.g., gadolinium chelates or iron oxides) to enable sensitive identification of numerous pathologies such as tumors^1^, vascular abnormalities^2^, and cardiac infarcts^3^ through local alterations in the tissue’s magnetic properties (T1 and T2 relaxation times). Clinical use of these contrast-enhanced MRI scans has further expanded as multiple contrast agents have been approved for specific clinical imaging applications (e.g., blood pool contrast agents^4^, hepatobiliary contrast agents^5^).

With the emergence of the field of molecular imaging, there has been a dramatic increase in the number of MRI contrast agents targeted to proteins^6–8^, cell receptors^9, 10^, and other molecular species^11, 12^. In addition, a number of activatable agents have been described that have different relaxivities based on the local tissue environment *in vivo* ^13, 14^. In a typical preclinical molecular MRI study, the T1 or T2 relaxation time (or MRI signal intensity) is measured dynamically before and after contrast agent administration allowing tracking of the agent’s distribution. These studies often incorporate non-targeted contrast agents (e.g., scrambled peptides) as controls to verify the *in vivo* molecular specificity of the targeting moiety. Due to the difficulty of previously-developed contrast enhanced MRI strategies to uniquely identify two MRI contrast agents administered simultaneously, the targeted and untargeted contrast agents must be studied in separate imaging sessions and likely in separate animal cohorts^15–17^. This significant limitation can result in experimental bias due to phenotypic variation. As such, the development of a “multi-color” MRI methodology to independently monitor simultaneously-administered targeted and control MRI contrast agents, and potentially multiple targeted MRI contrast agents, would significantly improve preclinical molecular MRI studies. This “multi-color” MRI capability would also provide a robust pathway for clinical translation of molecular MRI contrast agents by providing the capability to simultaneously compare the biodistribution of a molecular imaging agent with a conventional clinical MRI agent.

The primary limiting factor in the simultaneous assessment of multiple paramagnetic MRI contrast agents is that the unique identification of each agent is challenging. MRI contrast agents directly impact both the T1 and T2 relaxation times according to well-established concentration-dependent linear relationships to their magnetic relaxivities (r_1_ and r_2_) shown in equations (1a) and (1b) below^18^:

1a $$1/{\rm{T}}1=1/{\rm{T}}{1}_{0}+{{\rm{r}}}_{1{\rm{A}}}\times [{\rm{A}}]$$

1b $$1/{\rm{T}}2=1/{\rm{T}}{2}_{0}+{{\rm{r}}}_{2{\rm{A}}}\times [{\rm{A}}]$$

where [A] is the concentration of imaging agent A; T1_0_ and T2_0_ are the pre-contrast T1 and T2 relaxation times of the tissue; T1 and T2 are the post-contrast T1 and T2 relaxation times; and r_1A_ and r_2A_ are the magnetic relaxivities of contrast agent A. Therefore, while an individual MRI contrast agent is typically more sensitive to a particular relaxation parameter (i.e., Gd-chelates for enhancement in T1-weighted imaging acquisitions), each paramagnetic MRI contrast agent still impacts both the T1 and T2 relaxation times. This important factor limits the capability of MRI to independently assess simultaneously-administered contrast agents (e.g., a Gd-based T1 agent and an iron-based T2 agent).

The Magnetic Resonance Fingerprinting (MRF) methodology has recently been developed to simultaneously generate inherently co-registered T1 and T2 relaxation time maps in both patients^19–21^ and animal models^22, 23^. MRF uses a unique acquisition and quantification strategy that combines *a priori* acquisition parameter variation with a dictionary-based pattern matching algorithm to obtain quantitative assessments of multiple imaging parameters simultaneously. Importantly, MRF has been shown to provide quantitative T1 and T2 maps in 10–50 seconds per imaging slice providing the opportunity to dynamically generate quantitative maps of these two important MRI parameters simultaneously^24–27^. In this study, we demonstrate that the framework for simultaneous T1 and T2 assessments provided by MRF can be used to analytically quantify the local concentration of two different MRI contrast agents present at the same time. Herein, we describe a straightforward multiple contrast agent relaxation model (equations (2a) and (2b) in *METHODS)* that can be used in combination with the rapid, multi-parametric MRF strategy to independently calculate inherently co-registered concentration maps for two MRI contrast agents. These initial *in vitro* results represent a proof-of-concept study to: (1) validate the multiple contrast agent relaxation model using a 60 MHz magnetic relaxometer; and (2) demonstrate the application of the rapid MRF method to enable simultaneous calculation of concentration maps for two different paramagnetic MRI contrast agents on a clinical 3 T MRI scanner. Overall, these *in vitro* studies suggest an imaging framework for future *in vivo* MRI studies to simultaneously quantify multiple contrast agents.

## Results

### 60 MHz Relaxometry: Multiple Contrast Agent Relaxation Model Validation

**Figure 1:**
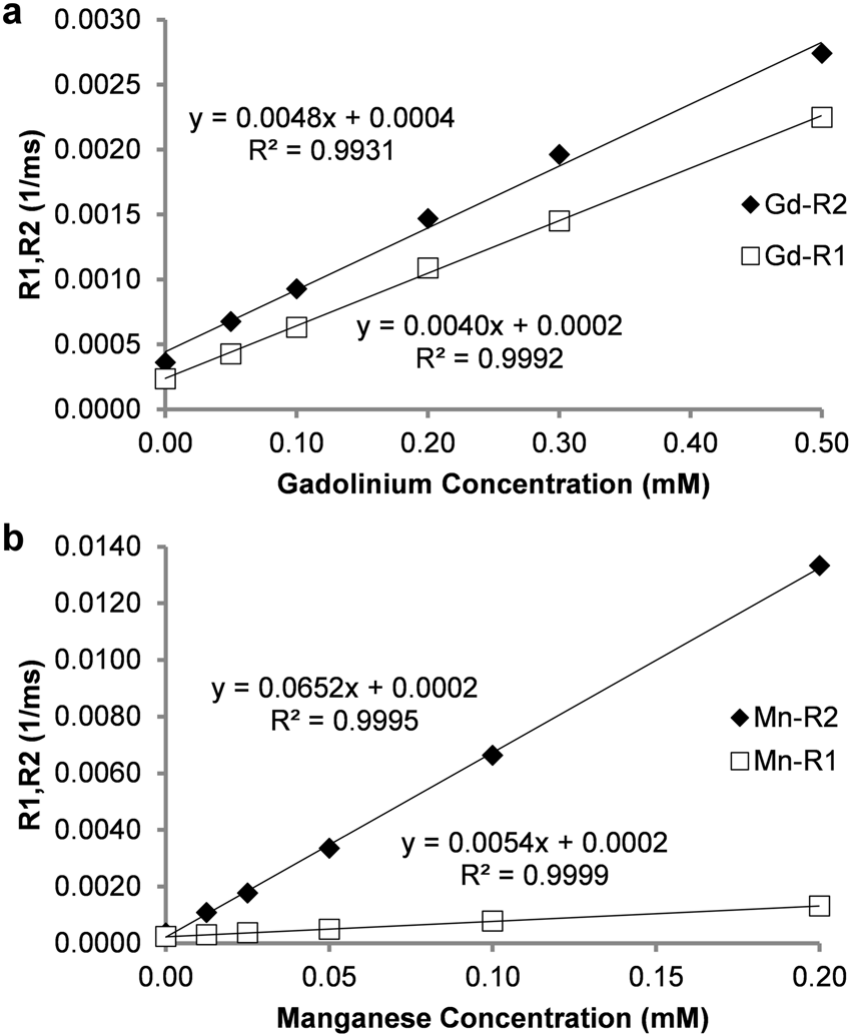
Relaxivity assessments for (**a**) gadolinium (Gd) and (**b**) manganese (Mn) contrast agents from a 60 MHz relaxometer using phantoms containing varying concentrations of a single contrast agent. Slopes of the fitted lines of R1, R2 vs. agent concentration (n = 6 for each agent) were used to determine the relaxivities (r_1_ and r_2_) of the two agents. Pearson correlations resulted in significant correlations of concentration vs. R1 and R2 for both contrast agents (R^2^ > 0.993, two-tailed probability p < 0.0001).

Figure 1 shows the individual magnetic relaxivity plots (R1 and R2 vs concentration) for the gadolinium and manganese contrast agent phantoms obtained from the 60 MHz relaxometer using inversion recovery spin-echo acquisitions for T1 relaxation time measurements followed sequentially by a Carr-Purcell-Meiboom-Gill (CPMG) acquisition for T2 relaxation time measurements. A list of *in vitro* phantoms and their respective concentrations of contrast agents is shown in Supplementary Table S1. The T1 measurements were repeated twice and the T2 assessments were repeated three times to ensure consistency. The relaxivity data resulted in significant linear correlations as expected from equations (1a) and (1b) (R^2^ > 0.993, p < 0.0001). The 60 MHz magnetic relaxivities at 37 °C for the gadolinium agent (slope of the linear correlation lines) were 0.0﻿040 mM^−1^ms^−1^ (r_1_) and 0.0﻿048 mM^−1^ms^−1^ (r_2_). The corresponding magnetic relaxivities for the manganese agent were 0﻿.0054 mM^−1^ms^−1^ (r_1_) and 0.﻿0652 mM^−1^ms^−1^ (r_2_). The magnetic relaxivity values for the 60 MHz relaxometer, as well as the relaxivities measured at 3 T, are shown in Table 1. The mean T1 and T2 relaxation times for the deionized water phantom with no contrast agent (deionized water alone) were 4250 ms (T1_0_) and 2760 ms (T2_0_), respectively. NMR relaxivity measurements are in reasonable agreement with previously reported results for both the Gd^28^ and Mn^29, 30^ contrast agents used here.

**Table 1:** Comparison of relaxivity measurements (r_1_, r_2_) in mM^−1^ms^−1^ for each contrast agent in deionized water at room temperature between 60 MHz*, 3 T spin echo (SE), and 3 T MRF.

Contrast Agent	r_1_ 60 MHz	r_2_ 60 MHz	r_1_ 3 T SE	r_1_ 3 T MRF	r_2_ 3 T SE	r_2_ 3 T MRF
Gd	﻿0.0040	﻿0.0048	﻿0.0051	﻿0.0056	﻿0.0060	﻿0.0076
Mn	﻿0.0054	﻿0.0652	﻿0.0068	﻿0.0067	﻿0.1079	﻿0.1144

^*^60 MHz measurements made at 37 °C.

**Figure 2:**
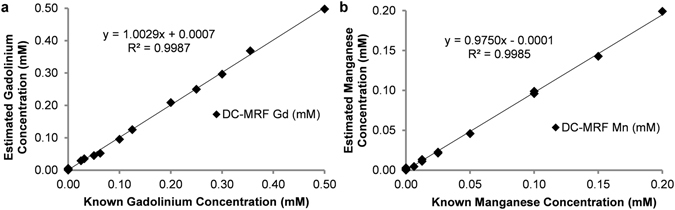
Pearson correlation plots of estimated (**a**) gadolinium (Gd), and (**b**) manganese (Mn) concentration versus known phantom concentrations (n = 17). Estimated concentrations were obtained from equations (3a) and (3b) for data obtained from a 60 MHz relaxometer. Note the significant correlation between the estimated and actual agent concentrations over all phantoms (Pearson Correlation: R^2^ > 0.998, two-tailed probability p < 0.0001).

Equations (3a) and (3b) (in *METHODS*) were then used to calculate the gadolinium and manganese concentrations for all of the phantoms (n = 17) using the measured T1 and T2 values for each *in vitro* sample, the calculated magnetic relaxivities for each contrast agent, and the “non-contrast” T1_0_ and T2_0_ values for the deionized water samples. Plots of the relaxometry-based concentration estimates against the known concentration in each phantom for both gadolinium and manganese chloride are shown in Fig. 2. Concentration estimates obtained from the 60 MHz relaxometry data shown in Fig. 2 were calculated for both the six samples containing both gadolinium and manganese contrast agents (Supplementary Table S1 Phantoms 11–16) as well as the ten samples containing either gadolinium or manganese used in the relaxometry analysis (Fig. 1, Supplementary Table S1 Phantoms 1–10). One additional sample containing only deionized water was also scanned (Supplementary Table S1 Phantom 17). Pure samples with a single contrast agent (either Gd or Mn) were analyzed to increase the number of concentrations calculated and to verify that the method returned a value of 0 if the agent was not present in solution. This comparison resulted in a significant linear correlation (Pearson Correlation: R^2^ > 0.998, two-tailed probability p < 0.0001) for both the gadolinium and manganese contrast agents. Importantly, the slopes of these correlations are both near unity (1.003 (Gd) and 0.975 (Mn), respectively) suggesting that the concentration estimates obtained from the multiple contrast agent model results in good agreement with the known concentrations. The results from only the six samples with both agents (Supplementary Table S1 Phantoms 11–16) are shown as a subset of the data separately (Supplementary Fig. S1). These initial relaxometric results demonstrate that the multiple contrast agent relaxation model shown in equations (2a) and (2b) is capable of providing accurate estimates for the concentration of gadolinium and manganese-based contrast agents. Importantly, these results appear to be consistent whether there is 0, 1, or 2 contrast agents in the phantom.

### 3 T DC-MRF: Simultaneous Assessment of Two Paramagnetic MRI Contrast Agents

**Figure 3:**
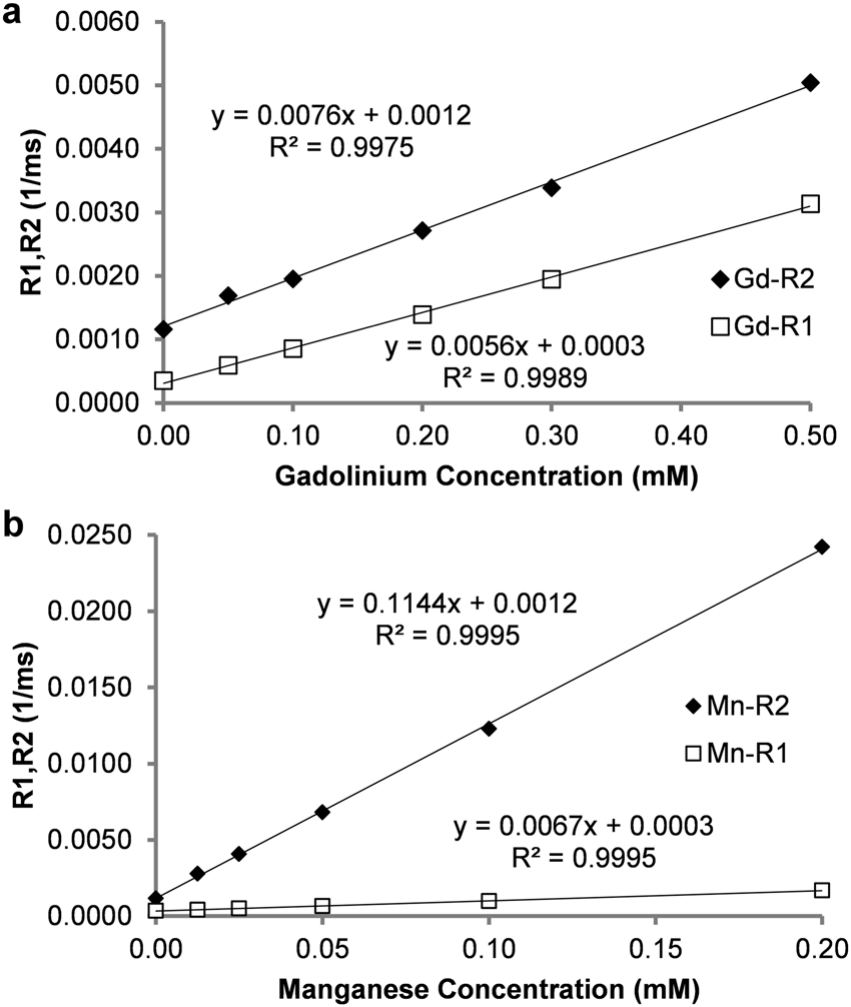
MRF-based relaxivity assessments for (**a**) gadolinium (Gd) and (**b**) manganese (Mn) contrast agents obtained on a 3 T MRI scanner using phantoms containing varying concentrations of a single contrast agent. Slopes of the fitted lines of R1, R2 vs. agent concentration (n = 6 for each agent) were used to determine the relaxivities (r_1_ and r_2_) of the two agents. Pearson correlations resulted in significant correlations of concentration vs. R1 and R2 for the two contrast agents (R^2^ ≥ 0.997, two-tailed probability p < 0.0001).

Similar to the relaxometry results above, T1 and T2 relaxation time assessments for gadolinium and manganese containing phantoms (n = 17) were obtained using the MRF method on a clinical 3 T MRI scanner at room temperature. Phantoms scanned contained the same contrast agent concentrations as for the NMR relaxometer experiments described above. This data was acquired using a FISP-MRF acquisition repeated 12 times following repositioning to measure average T1 and T2 values for each phantom. In contrast to the relaxometer studies above, the MRF-based T1 and T2 measurements were obtained simultaneously for all phantoms (n = 17). Figure 3 shows the individual magnetic relaxivity plots (R1 and R2 vs concentration) for the gadolinium and manganese contrast agents obtained from the MRF data averaged over the 12 repeats. The relaxivity data resulted in a significant linear correlation for both the gadolinium agent (Pearson Correlation: R^2^ ≥ 0.997, two-tailed probability p < 0.0001; r_1_ = 0.0056 mM^−1^ms^−1^_;_ r_2_ = 0.0076 mM^−1^ms^−1^) and the manganese agent (R^2^ ≥ 0.999, two-tailed probability p < 0.0001; r_1_ = 0.0067 mM^−1^ms^−1^_;_ r_2_ = 0.1144 mM^−1^ms^−1^). These MRF-based relaxivity values compared favorably to relaxivity values obtained from conventional inversion recovery and single-echo spin echo MRI experiments for both gadolinium (r_1_ = 0.0051 mM^−1^ms^−1^_;_ r_2_ = 0.0060 mM^−1^ms^−1^) and manganese (r_1_ = 0.0068 mM^−1^ms^−1^_;_ r_2_ = 0.1079 mM^−1^ms^−1^). Relaxivity results at 60 MHz and 3 T are summarized in Table 1. These relaxivity results also were in reasonable agreement with literature values^28, 29, 31^. DC-MRF was also used to obtain T1 and T2 relaxation times for the deionized water sample with no contrast agent (T1_0_ = 2897 ms; T2_0_ = 946 ms).

**Figure 4:**
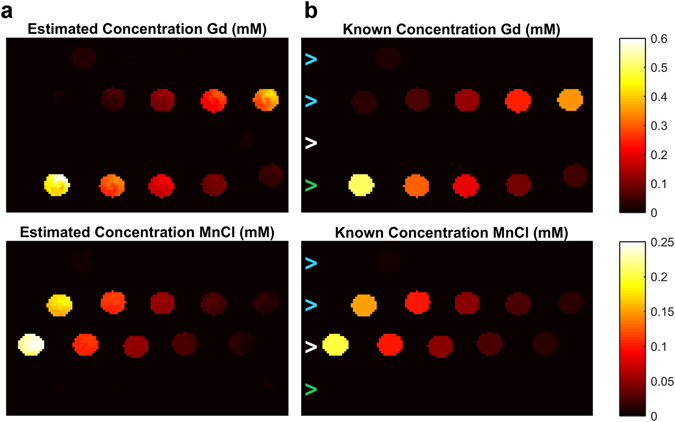
Maps of estimated gadolinium (Gd) and manganese (Mn) concentration from DC-MRF method (**a**). Simulated maps of known concentrations are shown for comparison (**b**). Note the general agreement between DC-MRF estimates and actual concentrations over a wide range of concentrations (n = 17). Note also the absence of signal from the vials containing only a single agent (bottom row of maps marked by green arrow contain only Gd, 3^rd^ row of maps marked by white arrow contain only Mn) indicating that the multiple contrast agent relaxation model and acquisition appears to be valid when the agents are used alone or in tandem (top two rows marked by blue arrows contain mixtures of both Gd and Mn contrast agents).

**Figure 5:**
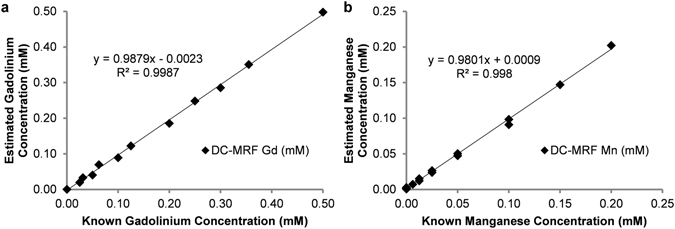
Pearson correlation plots of mean DC-MRF estimates for (**a**) gadolinium (Gd), and (**b**) manganese (Mn) concentration versus known phantom concentrations (n = 17). Mean DC-MRF concentration estimates were obtained from an ROI analysis of the MRF-based T1 and T2 relaxation time maps. The gadolinium and manganese concentrations were calculated for each of the MRF scans (n = 12) and averaged to calculate the mean DC-MRF concentration estimates shown ﻿in the plots. The mean DC-MRF concentration estimates resulted in a significant correlation over all phantoms (Pearson Correlations: R^2^ > 0.998, p < 0.0001). Note also that the slopes of the correlations are nearly equal to 1 (0.988 and 0.980 for Gd and Mn,﻿ respectively) indicative of limited bias in the DC-MRF results.

Representative DC-MRF maps of estimated gadolinium and manganese concentration calculated on a pixel-by-pixel basis from MRF-based T1 and T2 maps using equations (3a) and (3b) are shown in Fig. 4a for both the phantoms containing only gadolinium contrast agent (Supplementary Table S1 Phantoms 1–5; n = 5), only manganese contrast agent (Supplementary Table S1 Phantoms 6–10; n = 5), both gadolinium and manganese agents (Supplementary Table S1 Phantoms 11–16; n = 6), or solvent alone (Supplementary Table S1 Phantom 17; deionized water, n = 1). Theoretical maps of the known phantom concentrations are shown for comparison in Fig. 4b. The estimated gadolinium and manganese concentration maps are visually consistent with the known concentrations. A quantitative comparison of the mean DC-MRF concentration estimates from the region-of-interest analysis with the known concentrations are shown in Fig. 5 and resulted in significant correlations (Pearson Correlations: Gd: R^2^ = 0.9987, two-tailed probability p < 0.0001; Mn: R^2^ = 0.998, p < 0.0001). The mean DC-MRF concentrations were also in reasonable agreement with actual values as evidenced by the slopes of the correlation lines equal to 0.988 (Gd) and 0.980 (Mn), respectively. Results from the phantoms containing mixtures of both contrast agents are presented separately (Supplementary Fig. S2).

Mean and standard deviations of the gadolinium and manganese concentrations were calculated from the MRF-based concentration maps for the 6 phantoms containing both agents using an ROI analysis. The DC-MRF method resulted in significant differences among all samples for both the gadolinium concentration estimates (two-tailed unpaired Student’s t-test, p < 0.01) and the manganese concentration estimates (two-tailed unpaired Student’s t-test, p < 0.001). Overall, these results suggest that the DC-MRF methodology provides accurate and precise assessments of two paramagnetic MRI contrast agents at the same time.

## Discussion

In this initial report, we have demonstrated the capability of the Dual Contrast (DC)-MRF method to simultaneously measure the concentration of two paramagnetic MRI contrast agents. Herein, we present initial *in vitro* relaxometry measurements at 60 MHz to evaluate the proposed multiple contrast agent relaxation model for two paramagnetic MRI contrast agents (Equations (2a) and (2b)). We also show initial *in vitro* DC-MRF results on a clinical 3 T MRI scanner demonstrating the capability of DC-MRF to independently quantify the local concentration of two paramagnetic MRI contrast agents using simultaneously-measured T1 and T2 relaxation times. Overall, DC-MRF provides an imaging platform that can be used to independently monitor multiple paramagnetic MRI contrast agents with numerous clinical and preclinical molecular imaging applications.

We first validated the multiple contrast agent relaxation model described in equations (2a) and (2b). The significant correlations between the known phantom concentrations and the 60 MHz relaxometric estimates (R^2^ > 0.998, Fig. 2) as well as the 3 T DC-MRF estimates (R^2^ > 0.998, Figs. 4 and 5) suggests that this model is accurate for the concentration ranges of the two specific MRI contrast agents (Multihance**®**, gadobenate dimeglumine and MnCl_2_) used in this study. These results also demonstrate the ability of the model to accurately measure contrast agent concentrations at multiple field strengths. This straightforward linear relaxation model assumes that the two contrast agents impact the overall T1 and T2 relaxation times independently with minimal interactions between the two contrast agents. While this model may be expected to be valid for moderate agent concentrations, high local concentrations of one or both of the contrast agents could result in deviations from the linear model as the agents compete for interactions with the surrounding water molecules. Implicit in this model also is the assumption that the relaxivities (r_1_ and r_2_) are distinctly different for the two MRI contrast agents. Therefore, this relaxation model may have limitations if used to detect two MRI contrast agents with similar r_1_ and r_2_ values (e.g., gadopentetate dimeglumine and gadolinium-diethylenetriamine pentaacetic acid (Gd-DTPA)). Furthermore, multiple follow-on studies (*in vitro* and *in vivo*) will be needed to thoroughly explore the limitations of the proposed relaxation model. Regardless, these initial results demonstrate that the multiple contrast agent relaxation model can provide an analytical basis to determine the concentration of two different paramagnetic MRI contrast agents.

A key advantage of DC-MRF is that it provides the opportunity to simultaneously and dynamically detect multiple MRI contrast agents. Prior studies have attempted to detect multiple MRI contrast agents in a single scanning session using sequential administration of contrast agents^32^, ratiometric methods to detect the presence of activatable MRI agents^14^, and machine learning^33^. Other groups have utilized chemical exchange saturation transfer (CEST) MRI techniques which can have reduced sensitivity on low-field (≤ 3 T) MRI scanners^34–36^. It is important to note that these prior studies primarily incorporated sequential MRI assessments to separately assess the different MRI contrast agents. Sequential T1 and T2 measurements (or any other method that results in both T1 and T2 maps) could be used with equations (3a) and (3b) in place of MRF-based T1 and T2 assessments under the assumption that the concentration of the agent is not appreciably changing during the measurement time. Difficulty with this strategy arises because this assumption may or may not be valid based on the disease state and agents used. The ability of the MRF data to be acquired in as little as 10 seconds provides the opportunity to dynamically assess a wide variety of contrast agents regardless of the pharmacokinetics making it a more general solution with fewer required assumptions. Further, prior studies have shown that MRF is more temporally efficient than other rapid MRI techniques^37, 38^, and has been implemented on both clinical and preclinical MRI scanners. An additional benefit of the simultaneous measurement of T1 and T2 provided by MRF is these two relaxation time maps always being co-registered regardless of subject motion. This important feature allows the pixelwise concentration maps shown in Fig. 4b to be calculated without any mismatch errors or utilization of additional co-registration methodology. Therefore, DC-MRF may provide an adaptable, quantitative imaging framework to assess two MRI contrast agents simultaneously for a wide variety of imaging applications.

While these initial *in vitro* results suggest that DC-MRF can provide accurate assessments of two paramagnetic MRI contrast agents, *in vivo* imaging studies will be required to more fully evaluate the DC-MRF methodology. A schematic for an eventual *in vivo* DC-MRF implementation is shown in Supplementary Fig. S3. These *in vivo* DC-MRF experiments could be conducted similarly to conventional dynamic contrast enhanced MRI studies where images are collected before, during, and after administration of a contrast agent^39–42^. For *in vivo* DC-MRF, a pre-contrast MRF scan is performed to obtain baseline T1 and T2 maps (T1_0_ and T2_0_ in equations (2a) and (2b)). Both MRI contrast agents would then be administered simultaneously as a mixture (pseudo-color orange in syringe and tumor in Supplementary Fig. S3) during dynamic acquisition of MRF-based T1 and T2 maps. These inherently co-registered T1 and T2 maps would be then used with equations (3a) and (3b) to calculate *in vivo* concentration maps for each contrast agent (Agent A, red; Agent B, yellow; Supplementary Fig. S3).

Importantly, there are several challenges that must be overcome to utilize the DC-MRF methodology for *in vivo* experiments. First, MRF has been previously shown to be sensitive to inhomogeneities in both the B1 and B0 fields. As variation in B1 and B0 are expected to significantly increase for *in vivo* experiments, accurate B0 shimming and/or B1 corrections will be needed to avoid significant errors in T1 and T2 measurements^43^ as well as the calculated contrast agent concentrations. Additionally, *in vivo* relaxivities will likely be different from the *in vitro* relaxivities shown here due to complex molecular interactions experienced by the contrast agents *in vivo*. This may be particularly complicated as these interactions can vary considerably between normal tissues and pathologies. Addressing this important limitation will require careful *in vivo* relaxivity assessments and potentially validation using elemental analysis of excised tissues. The pre-contrast T1 and T2 relaxation times of the tissues and pathologies of interest may also pose specific challenges. For example, if the tissue of interest has a low T2 relaxation time before contrast administration (e.g., liver), resolving the concentration of the two agents may be problematic due to excessive T2 decay. In addition, *in vivo* studies may be impacted by multiple factors including partial volume effects, flow effect, and magnetization transfer and/or chemical exchange that may benefit from multi-exponential relaxation models instead of the simple mono-exponential models used here^44, 45^. Despite these challenges, DC-MRF offers a unique opportunity to expand the portfolio of *in vivo* contrast-enhanced MRI applications.

In addition to the need for follow-on *in vivo* studies, the DC-MRF results presented in this initial report have multiple areas for future exploration. As described above, the multiple contrast agent relaxation model described herein provides an analytical solution to independently quantify two MRI contrast agents. It is conceivable that DC-MRF could be applied for three or more contrast agents. However, in that case, equations (2a) and (2b) become underdetermined. As such, using the DC-MRF approach to differentiate three or more agents would require additional measurements and/or alternative numerical or machine learning^33^ approaches to resolve these agent concentrations accurately. As described above, another limitation of the DC-MRF methodology is the requirement for two agents with different relaxivities. In this initial proof-of-concept study, we used a clinical gadolinium agent and a manganese chloride based agent with substantial differences in r_1_ and r_2_ (Figs. 1 and 3). The minimum differences in relaxivity needed to reliably differentiate two contrast agents remains undetermined. Reduced relaxivity differences would also likely limit the ability of the DC-MRF methodology to detect small concentration changes. Additionally, these relaxivities are also known to change as a function of magnetic field strength. Therefore, *in vivo* validation studies would likely be required for each MRI field strength and for a variety of MRI contrast agents to explore the limitations of DC-MRF.

Although DC-MRF may have numerous applications, this new methodology is particularly well suited to support the development and eventual clinical translation of molecular MRI contrast agents. As described above, DC-MRF would provide the opportunity to detect both a molecularly-targeted contrast agent and a control non-targeted contrast agent at the same time in the same subject. The only constraint in achieving these simultaneous assessments using DC-MRF is that the two contrast agents must have different relaxivities. This constraint may require that the targeted and control contrast agents incorporate different lanthanides (e.g., gadolinium and dysprosium) in order to produce differential relaxivities while also retaining similar pharmacokinetic properties. A similar approach could also be used to allow multiple molecular imaging targets to be assessed simultaneously. For example, two targeted MRI contrast agents could be used to simultaneously track drug delivery and therapeutic impact in cancer treatment (e.g., a therapeutic agent^46^ labeled with gadolinium, and a second agent targeted to apoptosis^47^ labeled with dysprosium). Importantly, the DC-MRF methodology may also directly aid in the clinical translation of molecular imaging agents by allowing for the direct comparison between a conventional, non-targeted clinical MRI contrast agent (e.g., Multihance**®**) with a molecular imaging agent in a single patient. In this way, DC-MRF would provide the opportunity to efficiently establish the molecular specificity of the molecular contrast agent in heterogeneous human diseases.

In conclusion, we describe a new Dual Contrast - Magnetic Resonance Fingerprinting technique that can be used to independently quantify the local concentration of two paramagnetic MRI contrast agents administered simultaneously. These initial *in vitro* results validate the proposed multiple contrast agent relaxation model and demonstrate a new quantitative imaging methodology that can be used to simultaneously generate concentration maps for two different MRI contrast agents. Overall, these results suggest a new application for the MRF technology to provide quantitative assessments that lays the foundation for numerous clinical and preclinical multi-agent imaging applications.

## Methods

This section provides details on the validation of the multiple contrast agent relaxation model and the MRF acquisition used in combination to simultaneously estimate the concentration of two paramagnetic MRI contrast agents.

### Multiple Contrast Agent Relaxation Model

As described in the *INTRODUCTION* section, a single paramagnetic MRI contrast agent exhibits concentration-dependent T1 and T2 relaxation effects as described by equations (1a) and (1b), respectively. Herein, we are proposing a straightforward linear model to incorporate a second MRI contrast agent B as shown in equations (2a) and (2b):

2a $$1/{\rm{T}}1=1/{\rm{T}}{1}_{0}+{{\rm{r}}}_{1{\rm{A}}}\times [{\rm{A}}]+{{\rm{r}}}_{1{\rm{B}}}\times [{\rm{B}}]$$

2b $$1/{\rm{T}}2=1/{\rm{T}}{2}_{0}+{{\rm{r}}}_{2{\rm{A}}}\times [{\rm{A}}]+{{\rm{r}}}_{2{\rm{B}}}\times [{\rm{B}}]$$

where [B] is the concentration of agent B, and r_1B_ and r_2B_ are the magnetic relaxivities of contrast agent B. These equations suggest that if T1_0_, T2_0_, T1, and T2 are measured before and after simultaneous injection of two MRI contrast agents with known relaxivities, then these two equations have only two unknowns allowing for the direct analytical calculation of [A] and [B].

In this initial study, we tested the validity of this model using *in vitro* phantoms containing varying concentrations of gadolinium (Multihance**®**, gadobenate dimeglumine) and manganese (MnCl_2_,1 M Stock Solution, Sigma-Aldrich, #M1787) contrast agents either as single agents (i.e., Gd or Mn only) or as mixtures of the two agents (i.e., Gd and Mn combined) (concentrations in Supplementary Table S1). Gadolinium and manganese were chosen due to the wide clinical availability (Gd) and distinct relaxivity properties of the two agents. We first prepared serial dilutions of each contrast agent individually in deionized water to enable assessment of the individual relaxivities for each agent (r_1G_, r_2G_, r_1M_, r_2M_). A table explicitly listing the concentrations of the phantoms is provided online as Supplementary Table S1. For the gadolinium agent, the concentration was varied from 0.05 to 0.5 mM (Supplementary Table S1 Phantoms 1–5, n = 5). For the manganese agent, the concentration was varied from 0.0125 to 0.2 mM (Supplementary Table S1 Phantoms 6–10, n = 5). For both gadolinium and manganese a phantom of pure solvent was also analyzed (deionized water, Supplementary Table S1 Phantom 17). We then prepared mixtures of the two MRI contrast agents in the same solvent (Gd concentration range = 0.025 to 0.355 mM; Mn concentration range = 0.00625 to 0.15 mM; Supplementary Table S1 Phantoms 11–16, n = 6). 50 mL of each solution was prepared and served as a source for both the 60 MHz relaxometry and 3 T experiments.

To test the multiple contrast agent relaxation model in equations (2a) and (2b), each of the samples were transferred to 5 mm NMR tubes (Norell, 507-HP-7) and scanned on a Bruker Minispec 60 MHz relaxometer (Bruker Biospin, Billerica, MA). The relaxometer was used to obtain T1 and T2 relaxation time assessments for each sample using an inversion recovery spin echo technique (T1: 7 inversion times) and a multi-echo Carr-Purcell-Meiboom-Gill (CPMG) MRI acquisition (1,000–10,0000 echoes)^48^. All relaxometry experiments were conducted at 37 °C. The relaxometric measurements were repeated twice for T1 measurements and three times for T2 measurements to ensure accurate assessments. These repeated measures were averaged to obtain single T1 and T2 values for each *in vitro* phantom.

The resulting T1 and T2 values for each phantom were then used to calculate the agent concentration in each tube in a multi-step process. First, the T1 and T2 values for the solvent (deionized water) alone were measured and established as “pre-contrast” relaxation times (T1_0_ and T2_0_). Second, the phantoms containing the individual agents were analyzed to calculate the magnetic relaxivities of both agents (r_1G_, r_2G_, r_1M_, r_2M_) through a linear least-squares fit to the plot of R1 (1/T1 in ms^−1^; Equation (1a)) and R2 (1/T2 in ms^−1^; Equation (1b)) as a function of gadolinium or manganese concentration (in mM) using established methods. The slopes of the resulting fits were used as the magnetic relaxivities for each agent. From these relaxivity results, as well as the “pre-contrast” T1_0_ and T2_0_ values, the T1 and T2 relaxation times for each sample were used to analytically calculate the gadolinium and manganese contrast agent concentrations for each sample using equations (3a) and (3b). These equations were derived directly from the algebraic solution to equations (2a) and (2b) (with Gd = A and Mn = B):

3a $$[{\rm{G}}{\rm{d}}]=(({\rm{\Delta }}R2\times {{\rm{r}}}_{1{\rm{M}}})-({\rm{\Delta }}R1\times {{\rm{r}}}_{2{\rm{M}}}))/(({{\rm{r}}}_{2{\rm{G}}}\times {{\rm{r}}}_{1{\rm{M}}})-({{\rm{r}}}_{1{\rm{G}}}\times {{\rm{r}}}_{2{\rm{M}}}))$$

3b $$[{\rm{M}}{\rm{n}}]=({\rm{\Delta }}{\rm{R}}2-{(r}_{2{\rm{G}}}\times [{\rm{G}}{\rm{d}}])/{{\rm{r}}}_{2{\rm{M}}}$$

where ΔR1 = 1/T1–1/T1_0_, ΔR2 = 1/T2–1/T2_0_. The agent concentrations calculated from equations (3a) and (3b) were then compared with known concentrations in each phantom using Pearson correlations with a probability of p < 0.05 used as a determination for significance.

### *In Vitro* DC-MRF Assessments at 3 T

To determine the capability of the DC-MRF technique to provide quantitative imaging based assessments of contrast agent concentration, we obtained MRF-based T1 and T2 maps of the gadolinium-containing and manganese-containing phantoms evaluated in the relaxometry studies described above. The solutions in these phantoms were taken from the same source solutions as the NMR studies and placed into 15 mL centrifuge tubes (Fisher Scientific, S50712). All MRF studies were conducted on a Siemens Skyra 3 T MRI scanner (Siemens Healthineers, Erlangen, Germany). The MRF acquisition (Siemens Work-In-Progress #881v23) utilized a FISP acquisition kernel designed with *a priori* variation in both flip angle (FA) and repetition time (TR, baseline TR of 12 ms) to generate MRF signal evolution profiles sensitive to both T1 and T2 relaxation times^25^. The MRF method acquired 3000 images with time-varying contrast generated by the FA and TR variation. This FISP-MRF implementation included a non-selective inversion preparation (inversion time = 21 ms) immediately prior to the FISP-MRF image acquisitions to increase the sensitivity of the MRF signal evolution profiles to T1 relaxation times^22^. The MRF acquisition also incorporated undersampled spiral trajectories as described previously^25^. The acquisition time of one slice was 47 seconds with a FOV of 380 × 380 mm, an image matrix of 352 × 352, and a slice thickness of 5 mm. Measurement of the excitation (B1) field was incorporated to mitigate the effects of inhomogeneous B1 field on the T1 and T2 relaxation time estimates^43^.

A fundamental component of the MRF reconstruction is the development of a large dictionary of signal evolution profiles that are subsequently “matched” to the acquired MRF signal evolution profile for each imaging voxel using vector-based inner product comparisons. The MRF dictionary was created as described previously^24^ assuming a mono-exponential relaxation model. MRF image reconstruction as well as subsequent dictionary matching were performed on the Siemens Skyra 3 T MRI scanner. Generation of the quantitative MRF-based T1 and T2 relaxation time maps was attained by matching the acquired MRF profiles on a pixel-by-pixel basis to the MRF dictionary of simulated profiles from all logical combinations of T1 (10–100 ms, increment = 10 ms; 100–1000 ms, increment = 20 ms; 1000–2000 ms, increment = 40 ms; 2000–4500 ms, increment = 100 ms) and T2 (2–100 ms, increment = 2 ms; 100–150 ms, increment = 5 ms; 160–300 ms, increment = 10 ms; 300–800 ms, increment = 50 ms; 800–1600 ms, increment = 100 ms; 1600–3000 ms, increment = 200 ms). The T1 and T2 maps were exported for further offline processing in MATLAB (MathWorks, Natick, MA).

Mean T1 and T2 values for each phantom were obtained from the MRF maps using a region of interest (ROI) analysis. Similar to the relaxometric studies above, mean MRF-based T1 and T2 values for the sample with no contrast agent (deionized water only) was used as a measure of T1_0_ and T2_0_, respectively. The mean T1 and T2 relaxation times from the phantoms containing only a single agent were used to estimate the magnetic relaxivities for the gadolinium and manganese contrast agents. The T1 and T2 maps from the MRF acquisition were then used to calculate gadolinium and manganese concentration maps using the calculated 3 T relaxivities and equations (3a) and (3b), respectively. An ROI analysis of the maps was then used to calculate a mean gadolinium and manganese concentration value for each phantom. The mean DC-MRF concentration estimates were compared with known values using Pearson correlations. The MRF acquisition was repeated 12 times allowing for sample repositioning as well as scanner adjustments to test the capability of the DC-MRF method to statistically differentiate samples with both contrast agents.

Magnetic relaxivities were also obtained using conventional MRI assessments for comparison with the MRF-based relaxivity assessments. T1 relaxation time assessments were obtained with an inversion recovery spin echo acquisition (8 inversion times), and T2 relaxation times were obtained with a single-echo spin echo acquisition (8 echo times). Fitting was performed with the appropriate mono-exponential model generating T1 and T2 maps. Equations (1a) and (1b) were then used to calculate r_1_ and r_2_ for the gadolinium and manganese contrast agents, respectively.

### Statistical Analysis

Data were compared using Pearson correlation and unpaired, two-tailed Student’s t-tests when appropriate. In this study, p < 0.05 was used to establish statistical significance and the p-values are reported as less than the largest reasonable, round number. Repeated studies were performed on the same days after repositioning of the specimen and performing scanner adjustments including magnetic field shimming, radiofrequency excitation calibration, and receiver gain adjustments.

### Data Availability

The data and protocols used in the current study are available from the corresponding author on reasonable request.

## Electronic supplementary material

Supplementary Materials

## References

[1] Padhani, A. R. *et al*. Dynamic contrast enhanced MRI of prostate cancer: correlation with morphology and tumour stage, histological grade and PSA. *Clin. Radiol.* **55**, 99–109 (2000). 10.1053/crad.1999.0327 10657154

[2] Herborn, C. U. *et al*. Comprehensive time-resolved MRI of peripheral vascular malformations. *Am. J. Roentgenol.* **181**, 729–735 (2003). 10.2214/ajr.181.3.1810729 12933470

[3] Lima, J. A. C. *et al*. Regional heterogeneity of human myocardial infarcts demonstrated by contrast-enhanced MRI. *Circulation* **92**, 1117–1125 (1995). 10.1161/01.cir.92.5.1117 7648655

[4] Bock, J. *et al*. 4D phase contrast MRI at 3 T: effect of standard and blood-pool contrast agents on SNR, PC-MRA, and blood flow visualization. *Magn. Reson. Med.* **63**, 330–338 (2010). 10.1002/mrm.22199 20024953

[5] Reimer, P., Schneider, G. & Schima, W. Hepatobiliary contrast agents for contrast-enhanced MRI of the liver: properties, clinical development and applications. *Eur. Radiol.* **14**, 559–578 (2004). 10.1007/s00330-004-2236-1 14986050

[6] Pu, F. *et al*. Prostate-specific membrane antigen targeted protein contrast agents for molecular imaging of prostate cancer by MRI. *Nanoscale* **8**, 12668; 10.1053/crad.1999.0327 (2016). 10.1039/c5nr09071g PMC5528195 26961235

[7] Zhou, Z., Han, Z. & Lu, Z. R. A targeted nanoglobular contrast agent from host-guest self-assembly for MR cancer molecular imaging. *Biomaterials* **85**, 168–179 (2016). 10.1016/j.biomaterials.2016.02.002 PMC5412079 26874280

[8] Kitagawa, T. *et al*. RGD targeting of human ferritin iron-oxide nanoparticles enhances *in vivo* molecular MRI of experimental aortic aneurysms. *J. Magn. Reson. Imaging* **45**, 1144–1153 (2016). 10.1002/jmri.25459 PMC5352511 27689830

[9] Liu, X. *et al*. MRI contrast agent for targeting glioma: interleukin-13 labeled liposome encapsulating gadolinium-DTPA. *Neuro. Oncol.* **18**, 691–699 (2016). 10.1093/neuonc/nov263 PMC4827043 26519740

[10] Li, T. *et al*. A new interleukin-13 amino-coated gadolinium metallofullerene nanoparticle for targeted MRI detection of glioblastoma tumor cells. *J. Am. Chem. Soc.* **137**, 7881–7888 (2015). 10.1021/jacs.5b03991 26022213

[11] Abakumova, T. *et al*. Connexin 43-targeted T1 contrast agent for MRI diagnosis of glioma. *Contrast Media Mol. Imaging* **11**, 15–23 (2016). 10.1002/cmmi.1653 26265140

[12] Zhou, Z. *et al*. MRI detection of breast cancer micrometastases with a fibronectin-targeting contrast agent. *Nat. Commun.* **6**, 7984; doi:10.1038/ncomms8984 (2015). 10.1038/ncomms8984 PMC4557274 26264658

[13] Gale, E. M., Jones, C. M., Ramsay, I., Farrar, C. T. & Caravan, P. A Janus chelator enables biochemically responsive MRI contrast with exceptional dynamic range. *J. Am. Chem. Soc.* **138**, 15861–15864 (2016). 10.1021/jacs.6b10898 PMC5328420 27960350

[14] Catanzaro, V. *et al*. A R2p/R1p ratiometric procedure to assess matrix metalloproteinase-2 activity by magnetic resonance imaging. *Angew. Chemie - Int. Ed.* **52**, 3926–3930 (2013). 10.1002/anie.201209286 23450786

[15] Kim, K. S., Park, W., Hu, J., Bae, Y. H. & Na, K. A cancer-recognizable MRI contrast agents using pH-responsive polymeric micelle. *Biomaterials* **35**, 337–343 (2014). 10.1016/j.biomaterials.2013.10.004 24139764

[16] Fuchs, B. C. *et al*. Molecular MRI of collagen to diagnose and stage liver fibrosis. *J. Hepatol.* **59**, 992–998 (2013). 10.1016/j.jhep.2013.06.026 PMC3805694 23838178

[17] Kircher, M. F. *et al*. A brain tumor molecular imaging strategy using a new triple-modality MRI-photoacoustic-Raman nanoparticle. *Nat. Med.* **18**, 829–834 (2012). 10.1038/nm.2721 PMC3422133 22504484

[18] Wood, M. L. & Hardy, P. A. Proton relaxation enhancement. *J. Magn. Reson. Imaging* **3**, 149–156 (1993). 10.1002/jmri.1880030127 8428082

[19] Assländer, J., Glaser, S. J. & Hennig, J. Pseudo steady-state free precession for MR-Fingerprinting. *Magn. Reson. Med.* **77**, 1151–1161 (2016). 10.1002/mrm.26202 27079826

[20] Hamilton, J. I. *et al*. MR fingerprinting for rapid quantification of myocardial T1, T2, and proton spin density. *Magn. Reson. Med.* **77**, 1446–1459 (2016). 10.1002/mrm.26216 PMC5045735 27038043

[21] Cauley, S. F. *et al*. Fast group matching for MR fingerprinting reconstruction. *Magn. Reson. Med.* **74**, 523–528 (2014). 10.1002/mrm.25439 PMC4700821 25168690

[22] Gao, Y. *et al*. Preclinical MR fingerprinting (MRF) at 7 T: effective quantitative imaging for rodent disease models. *NMR Biomed.* **28**, 384–394 (2015). 10.1002/nbm.3262 PMC4396690 25639694

[23] Buonincontri, G. & Sawiak, S. J. MR fingerprinting with simultaneous B1 estimation. *Magn. Reson. Med.* **76**, 1127–1135 (2016). 10.1002/mrm.26009 PMC5061105 26509746

[24] Ma, D. *et al*. Magnetic resonance fingerprinting. *Nature* **495**, 187–192 (2013). 10.1038/nature11971 PMC3602925 23486058

[25] Jiang, Y., Ma, D., Seiberlich, N., Gulani, V. & Griswold, M. MR fingerprinting using fast imaging with steady state precession (FISP) with spiral readout. *Magn. Reson. Med.* **74**, 1621–1631 (2015). 10.1002/mrm.25559 PMC4461545 25491018

[26] Ye, H. *et al*. Accelerating magnetic resonance fingerprinting (MRF) using t-blipped simultaneous multislice (SMS) acquisition. *Magn. Reson. Med.* **75**, 2078–2085 (2016). 10.1002/mrm.25799 PMC4673043 26059430

[27] Cloos, M. A. *et al*. Multiparametric imaging with heterogeneous radiofrequency fields. *Nat. Commun.* **7**, 12445; doi:10.1038/ncomms12445 (2016). 10.1038/ncomms12445 PMC4990694 27526996

[28] Rohrer, M., Bauer, H., Mintorovitch, J., Requardt, M. & Weinmann, H.-J. Comparison of magnetic properties of MRI contrast media solutions at different magnetic field strengths. *Invest. Radiol.* **40**, 715–724 (2005). 10.1097/01.rli.0000184756.66360.d3 16230904

[29] Caravan, P., Farrar, C., Frullano, L. & Uppal, R. Contrast agents. *Contrast Media Mol. Imaging* **4**, 89–100 (2009). 10.1002/cmmi.267 PMC3995751 19177472

[30] Lauffer, R. B. Paramagnetic metal complexes as water proton relaxation agents for NMR imaging: theory and design. *Chem. Rev.* **87**, 901–927 (1987).

[31] Nofiele, J. T. & Cheng, H. L. M. Ultrashort echo time for improved positive-contrast manganese-enhanced MRI of cancer. *PLoS One* **8**, e58617; doi:10.1371/journal.pone.0058617 (2013). 10.1371/journal.pone.0058617 PMC3587583 23484042

[32] Kubaska, S., Sahani, D. V., Saini, S., Hahn, P. F. & Halpern, E. Dual contrast enhanced magnetic resonance imaging of the liver with superparamagnetic iron oxide followed by gadolinium for lesion detection and characterization. *Clin. Radiol.* **56**, 410–415 (2001). 10.1053/crad.2000.0673 11384141

[33] Hung, A. H., Lilley, L. M., Hu, F., Harrison, V. S. R. & Meade, T. J. Magnetic barcode imaging for contrast agents. *Magn. Reson. Med.* **77**, 970–978 (2017). 10.1002/mrm.26175 PMC5055837 27062518

[34] Ali, M. M., Liu, G., Shah, T., Flask, C. A. & Pagel, M. D. Using two chemical exchange saturation transfer magnetic resonance imaging contrast agents for molecular imaging studies. *Acc. Chem. Res.* **42**, 915–924 (2009). 10.1021/ar8002738 PMC6010180 19514717

[35] Fernández-Cuervo, G., Sinharay, S. & Pagel, M. D. A catalyCEST MRI contrast agent that can simultaneously detect two enzyme activities. *ChemBioChem* **17**, 383–387 (2016). 10.1002/cbic.201500586 PMC4814164 26693680

[36] Hingorani, D. V. *et al*. A single diamagnetic catalyCEST MRI contrast agent that detects cathepsin B enzyme activity by using a ratio of two CEST signals. *Contrast Media Mol. Imaging* **11**, 130–138 (2016). 10.1002/cmmi.1672 PMC4882611 26633584

[37] Schmitt, P. *et al*. Inversion recovery TrueFISP: quantification of T1, T2, and spin density. *Magn. Reson. Med.* **51**, 661–667 (2004). 10.1002/mrm.20058 15065237

[38] Deoni, S. C. L., Peters, T. M. & Rutt, B. K. High-resolution T1 and T2 mapping of the brain in a clinically acceptable time with DESPOT1 and DESPOT2. *Magn. Reson. Med.* **53**, 237–241 (2005). 10.1002/mrm.20314 15690526

[39] Herrmann, K. *et al*. Dynamic quantitative T1 mapping in orthotopic brain tumor xenografts. *Transl. Oncol.* **9**, 147–154 (2016). 10.1016/j.tranon.2016.02.004 PMC4833967 27084431

[40] Towner, R. A. *et al*. *In vivo* detection of c-Met expression in a rat C6 glioma model. *J. Cell. Mol. Med.* **12**, 174–186 (2008). 10.1111/j.1582-4934.2008.00220.x PMC3823479 18194445

[41] Ingrisch, M. *et al*. Quantification of perfusion and permeability in multiple sclerosis. *Invest. Radiol.* **47**, 252–258 (2012). 10.1097/RLI.0b013e31823bfc97 22373532

[42] Taheri, S., Gasparovic, C., Shah, N. J. & Rosenberg, G. A. Quantitative measurement of blood-brain barrier permeability in human using dynamic contrast-enhanced MRI with fast T1 mapping. *Magn. Reson. Med.* **65**, 1036–1042 (2011). 10.1002/mrm.22686 PMC4950947 21413067

[43] Ma, D. *et al*. Slice profile and B_1_ corrections in 2D magnetic resonance fingerprinting. *Magn. Reson. Med*.; doi:10.1002/mrm.26580 (2017). 10.1002/mrm.26580 PMC5505861 28074530

[44] Stanisz, G. J. & Henkelman, R. M. Gd-DTPA relaxivity depends on macromolecular content. *Magn. Reson. Med.* **44**, 665–667 (2000). 10.1002/1522-2594(200011)44:5<665::aid-mrm1>3.0.co;2-m 11064398

[45] Dula, A. N., Gochberg, D. F., Valentine, H. L., Valentine, W. M. & Does, M. D. Multiexponential T2, magnetization transfer, and quantitative histology in white matter tracts of rat spinal cord. *Magn. Reson. Med.* **63**, 902–909 (2010). 10.1002/mrm.22267 PMC2852261 20373391

[46] Beg, M. S., Mohapatra, J., Pradhan, L., Patkar, D. & Bahadur, D. Porous Fe3O4-SiO2 core-shell nanorods as high-performance MRI contrast agent and drug delivery vehicle. *J. Magn. Magn. Mater.* **428**, 340–347 (2017).

[47] Hiller, K.-H., Waller, C., Nabrendorf, M., Bauer, W. R. & Jakob, P. M. Assessment of cardiovascular apoptosis in the isolated rat heart by magnetic resonance molecular imaging. *Mol. Imaging* **5**, 115–121 (2006). 16954025

[48] Majumdar, S., Zoghbi, S., Pope, C. F. & Gore, J. C. Quantitation of MR relaxation effects of iron oxide particles in liver and spleen. *Radiology* **169**, 653–658 (1988). 10.1148/radiology.169.3.3186986 3186986

