# Supplementary material for: Dual Contrast - Magnetic Resonance Fingerprinting (DC-MRF): A Platform for Simultaneous Quantification of Multiple MRI Contrast Agents
Source: Sci Rep. 2017 Aug 16;7:8431. doi: 10.1038/s41598-017-08762-9 (PMC5559598; doi:10.1038/s41598-017-08762-9)
Supplement: Supplementary file 1 — Supplementary Materials [file 41598_2017_8762_MOESM1_ESM.pdf]

Dual Contrast - Magnetic Resonance Fingerprinting (DC-MRF): A Platform for Simultaneous Quantification of Multiple MRI Contrast Agents

Christian E. Anderson[1,2], Shannon B. Donnola[1], Yun Jiang[1], Joshua Batesole[1], Rebecca Darrah[3,4], Mitchell L. Drumm[4,5], Susann M. Brady-Kalnay[6,7], Nicole F. Steinmetz[1,2,8,9,10], Xin Yu[2,11], Mark A. Griswold[1,2], Chris A. Flask[1,2,5*]

[1]Department of Radiology, Case Western Reserve University, Cleveland, OH.
[2]Department of Biomedical Engineering, Case Western Reserve University, Cleveland, OH.
[3]Frances Payne Bolton School of Nursing, Case Western Reserve University, Cleveland, OH.
[4]Department of Genetics and Genome Sciences, Case Western Reserve University, Cleveland, OH.
[5]Department of Pediatrics, Case Western Reserve University, Cleveland, OH.
[6]Department of Molecular Biology and Microbiology, Case Western Reserve University, Cleveland, OH.
[7]Department of Neurosciences, Case Western Reserve University, Cleveland, OH.
[8]Department of Materials Science and Engineering, Case Western Reserve University, Cleveland, OH.
[9]Department of Macromolecular Science and Engineering, Case Western Reserve University, Cleveland, OH.
[10]Division of General Medicine-Oncology, Case Western Reserve University, Cleveland, OH.
[11]Department of Physiology and Biophysics, Case Western Reserve University, Cleveland, OH.
*Corresponding Author

This file contains supplementary figures for *Dual Contrast – Magnetic Resonance Fingerprinting: A Platform for Simultaneous Quantification of Multiple MRI Contrast Agents.* Each figure is on its own page.

Supplementary Table S1. In Vitro Phantom Concentrations. 5 Gadolinium-Only Phantoms (#1-5), 5 Manganese-Only Phantoms (#6-10), 6 Phantoms with Both Contrast Agent (#11-16), and one Solvent-Only Phantom (#17, deionized water).

Phantom	Gadolinium Concentration (mM)	Manganese Concentration (mM)
1	0.5	0
2	0.3	0
3	0.2	0
4	0.1	0
5	0.05	0
6	0	0.2
7	0	0.1
8	0	0.05
9	0	0.025
10	0	0.0125
11	0.03125	0.15
12	0.0625	0.1
13	0.125	0.05
14	0.25	0.025
15	0.355	0.0125
16	0.025	0.00625
17	0	0

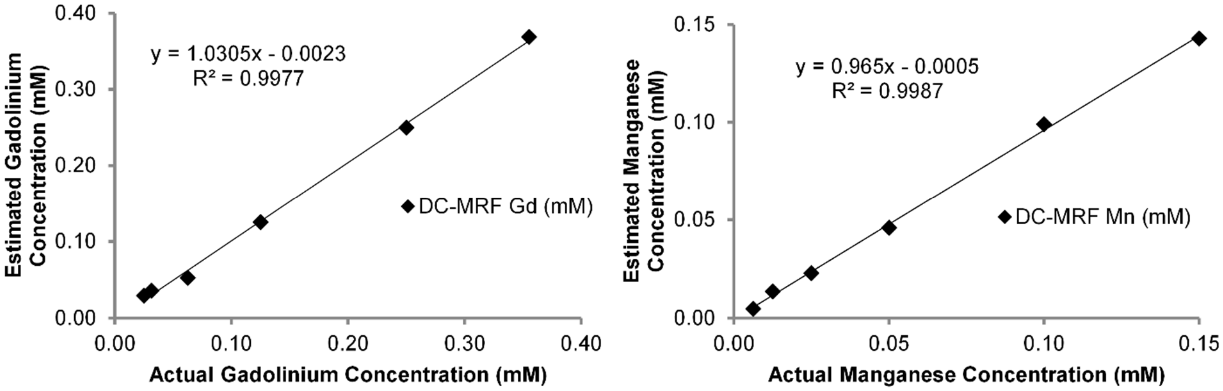

Supplementary Figure S1. Pearson correlation plots of mean DC-MRF estimates for Gd (left) and Mn (right) concentrations against known concentrations at 60 MHz from phantoms containing mixtures of both MRI contrast agents (Supplementary Table S1 Phantoms 11-16; n=6). Concentrations were estimated from the 60 MHz measurements of T1 and T2 and using equations (3a) and (3b). These data are a subset of the data shown in Figure 2.

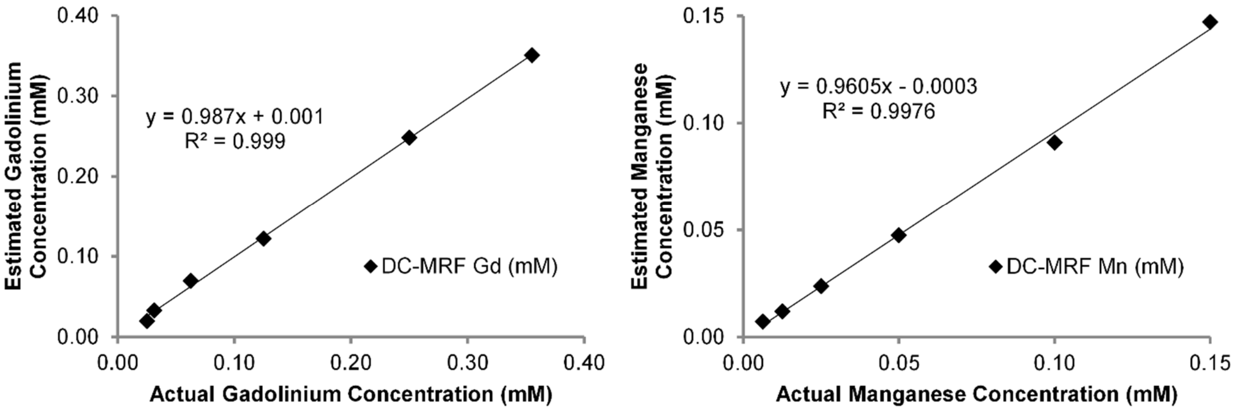

Supplementary Figure S2. Pearson correlation plots of mean DC-MRF estimates for Gd (left) and Mn (right) concentrations against known concentrations at 3T from phantoms containing mixtures of both contrast agents (Supplementary Table S1 Phantoms 11-16; n=6). Concentrations were estimated using equations (3a) and (3b) and mean T1 and T2 values obtained from MRF-based maps. These data are a subset of the data shown in Figure 5.

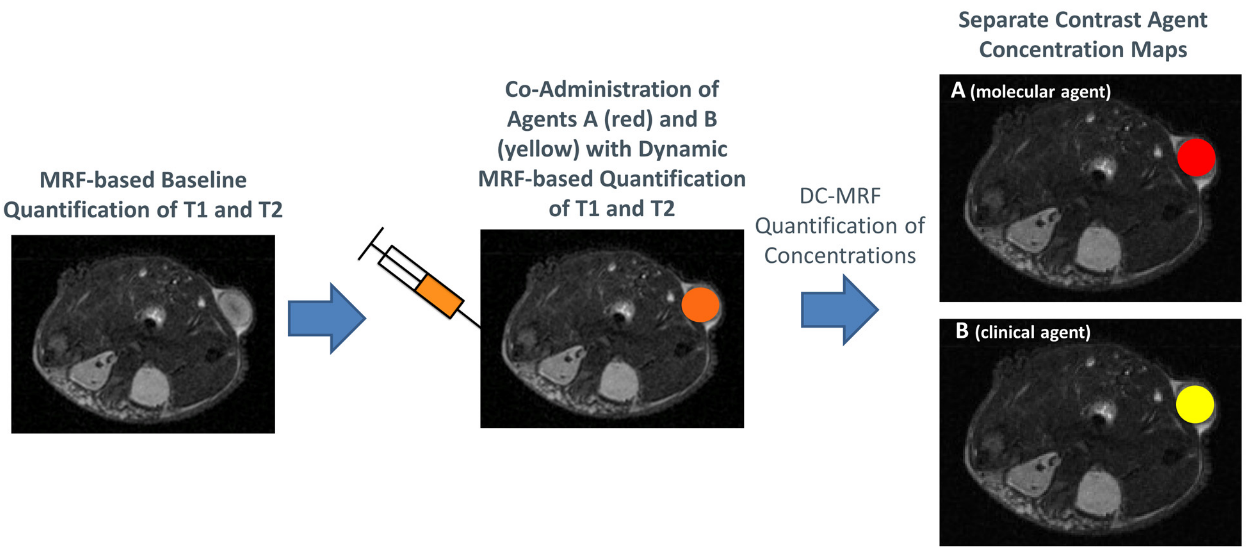

Supplementary Figure S3. Proposed workflow for in vivo DC-MRF application. This workflow describes how two agents (Agent A, red; Agent B, yellow) could be applied and quantified in vivo. A baseline MRF scan is first performed to provide pre-contrast T1 and T2 relaxation time maps ($T1_0$ and $T2_0$). After the baseline scans, a solution containing a mixture of two contrast agents is injected (pseudo-color orange in syringe and tumor) while MRF-based T1 and T2 maps are dynamically collected. These T1 and T2 maps can be used in the DC-MRF framework to yield pixel-wise calculations of ion concentration resulting in maps of each individual agent using Equations (3a) and (3b). This process assumes that the in vivo relaxivities (r_1,r_2) of the two MRI contrast agents have already been determined.
